# Crystal structure of 7-bromo-2-(3-fluoro­phen­yl)-1-(methyl­sulfin­yl)naphtho[2,1-*b*]furan

**DOI:** 10.1107/S160053681401808X

**Published:** 2014-08-13

**Authors:** Hong Dae Choi, Uk Lee

**Affiliations:** a Department of Chemistry, Dongeui University, San 24 Kaya-dong, Busanjin-gu, Busan 614-714, Republic of Korea; b Department of Chemistry, Pukyong National University, 599-1 Daeyeon 3-dong, Nam-gu, Busan 608-737, Republic of Korea

**Keywords:** crystal structure, naphtho­furan, 3-fluoro­benzene, C—H⋯O inter­actions, C—Br⋯π inter­actions

## Abstract

In the title compound, C_19_H_11_BrFO_2_S, the dihedral angle between the plane of the naphtho­furan ring system [r.m.s. deviation = 0.043 (2) Å] and that of the 3-fluoro­benzene ring is 39.32 (8)°. In the crystal, mol­ecules are linked by C—H⋯O and C—Br⋯π [3.835 (1) Å] inter­actions into stacks along the *c* axis, forming a three-dimensional network. The F atom is disordered over two positions, with site-occupancy factors of 0.851 (3) and 0.149 (3).

## Related literature   

For the pharmacological activities of compounds containing a naphtho­furan ring, see: Debnath *et al.* (1993 ▶); Einhorn *et al.* (1984 ▶); Hranjec *et al.* (2003 ▶); Mahadevan & Vaidya (2003 ▶). For the fluorescence properties of compounds having a naphtho­furan skeleton, see: Piloto *et al.* (2005 ▶). For the synthesis of the starting material 7-bromo-2-(3-fluoro­phen­yl)-1-(methyl­sulf­an­yl)naphtho­[2,1-*b*]furan, see: Choi *et al.* (1999 ▶). For a related structure, see: Choi *et al.* (2013 ▶).
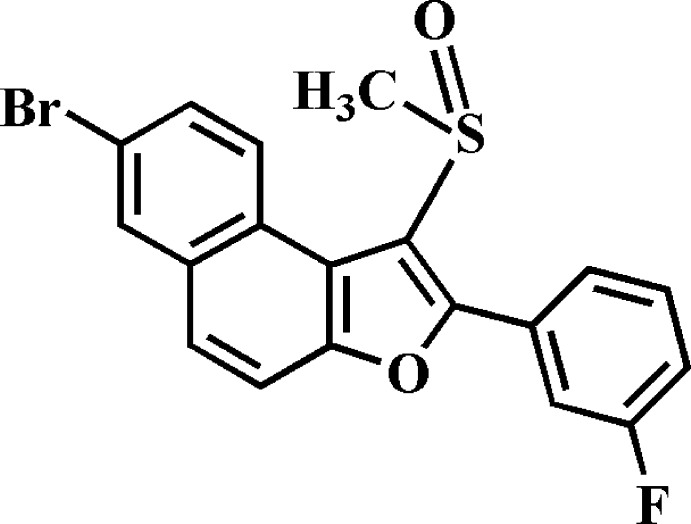


## Experimental   

### Crystal data  

C_19_H_12_BrFO_2_S
*M* *_r_* = 403.26
Monoclinic, 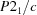

*a* = 6.1340 (1) Å
*b* = 23.0602 (5) Å
*c* = 10.8806 (2) Å
β = 91.166 (1)°
*V* = 1538.76 (5) Å^3^
*Z* = 4
Mo *K*α radiation
μ = 2.83 mm^−1^
*T* = 173 K
0.74 × 0.45 × 0.38 mm

### Data collection  

Bruker SMART APEXII CCD diffractometer
Absorption correction: multi-scan (*SADABS*; Bruker, 2009 ▶) *T*_min_ = 0.229, *T*_max_ = 0.413
14935 measured reflections
3834 independent reflections
3033 reflections with *I* > 2σ(*I*)
*R*_int_ = 0.047

### Refinement  

*R*[*F* ^2^ > 2σ(*F* ^2^)] = 0.037
*wR*(*F* ^2^) = 0.098
*S* = 1.04
3834 reflections
228 parameters
14 restraints
H-atom parameters constrained
Δρ_max_ = 0.47 e Å^−3^
Δρ_min_ = −0.91 e Å^−3^

### 

Data collection: *APEX2* (Bruker, 2009 ▶); cell refinement: *SAINT* (Bruker, 2009 ▶); data reduction: *SAINT*; program(s) used to solve structure: *SHELXS97* (Sheldrick, 2008 ▶); program(s) used to refine structure: *SHELXL97* (Sheldrick, 2008 ▶); molecular graphics: *ORTEP-3 for Windows* (Farrugia, 2012 ▶) and *DIAMOND* (Brandenburg, 1998 ▶); software used to prepare material for publication: *SHELXL97*.

## Supplementary Material

Crystal structure: contains datablock(s) I. DOI: 10.1107/S160053681401808X/tk5335sup1.cif

Structure factors: contains datablock(s) I. DOI: 10.1107/S160053681401808X/tk5335Isup2.hkl

Supporting information file. DOI: 10.1107/S160053681401808X/tk5335Isup3.cml

**Fig. 1.**
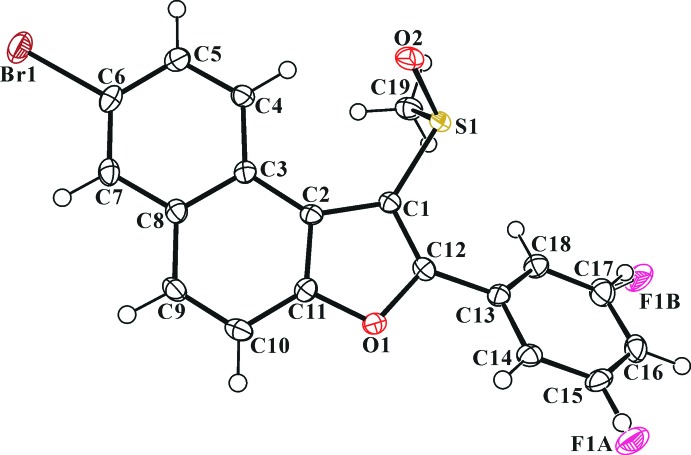
The molecular structure of the title compound with the atom numbering scheme. Displacement ellipsoids are drawn at the 50% probability level. H atoms are presented as small spheres of arbitrary radius. The F atom of the 3-fluorobenzene ring is disordered over two positions with site occupancy factors, from refinement of 0.851 (3) (part A) and 0.149 (3) (part B).

. DOI: 10.1107/S160053681401808X/tk5335fig1.tif
The mol­ecular structure of the title compound with the atom numbering scheme. Displacement ellipsoids are drawn at the 50% probability level. H atoms are presented as small spheres of arbitrary radius. The F atom of the 3-fluoro­benzene ring is disordered over two positions with site occupancy factors, from refinement of 0.851 (3) (part A) and 0.149 (3) (part B).

**Fig. 2.**
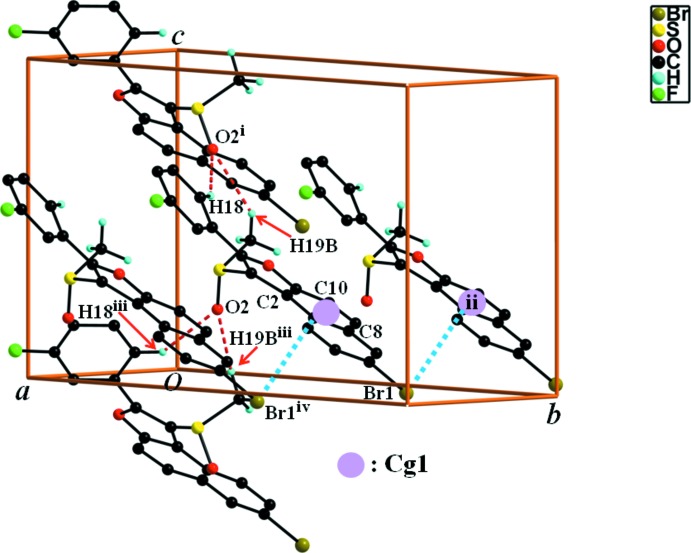
A view of the C—H···O and C—Br···π interactions (dotted lines) in the crystal structure of the title compound. H atoms non-participating in hydrogen-bonding are omitted for clarity [Symmetry codes: (i) x, - y + 1/2, z + 1/2; (ii) x - 1, y, z; (iii) x, - y + 1/2, z - 1/2].

Click here for additional data file.x y z x y z x y z . DOI: 10.1107/S160053681401808X/tk5335fig2.tif
A view of the C—H⋯O and C—Br⋯π inter­actions (dotted lines) in the crystal structure of the title compound. H atoms non-participating in hydrogen-bonding are omitted for clarity [Symmetry codes: (i) *x*, − *y* + 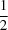, *z* + 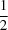; (ii) *x* − 1, *y*, *z*; (iii) *x*, − *y* + 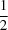, *z* − 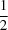].

CCDC reference: 1018271

Additional supporting information:  crystallographic information; 3D view; checkCIF report

## Figures and Tables

**Table 1:** Hydrogen-bond geometry (Å, °)

*D*—H⋯*A*	*D*—H	H⋯*A*	*D*⋯*A*	*D*—H⋯*A*
C18—H18⋯O2^i^	0.95	2.48	3.260 (3)	139
C19—H19*B*⋯O2^i^	0.98	2.56	3.387 (3)	142

Symmetry code: (i) 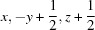.

## supplementary crystallographic information

### S1. Structural commentary

Many compounds involving a naphtho­furan moiety show potent biological activities such as anti­bacterial, anti­tumor, anthelmintic and mutagenic properties (Debnath *et al.*, 1993, Einhorn *et al.*, 1984, Hranjec *et al.*, 2003, Mahadevan *et al.*, 2003). These naphtho­furan derivatives are known about their fluorescence properties and potential utility as suitable fluorescent makers (Piloto *et al.*, 2005). As a part of our ongoing project of 7-bromo-2-aryl-1-(methyl­sulfinyl)­naphtho­[2,1-*b*]furan derivatives containing 4-methyl­phenyl substituent in 2-position (Choi *et al.*, 2013), we report herein on the crystal structure of the title compound.

In the title molecule (Fig. 1), the naphtho­[2,1-*b*]furan unit is essentially planar, with a mean deviation of 0.043 (2) Å from the least-squares plane defined by the thirteen constituent atoms. The 3-fluoro­phenyl ring is essentially planar, with a mean deviation of 0.008 (2) Å from the least-squares plane defined by the six constituent atoms. In the 3-fluoro­phenyl ring, the F atom is disordered over two positions with site-occupancy factors, from refinement, of 0.851 (3) (part A) and 0.149 (3) (part B). The dihedral angle formed by the naphtho­[2,1-*b*b]furan ring system and the 3-fluoro­phenyl ring is 39.32 (8)°. In the crystal structure (Fig. 2), molecules are linked by C—H···O hydrogen bonds (Table 1) and C6—Br1···π inter­actions between the bromine atom and the central benzene ring of a neighbouring molecule with a Br1···Cg1^ii^ being 3.835 (1) Å (Cg1 is the centroid of the C2/C3/C8/C9/C10/C11 benzene ring), into stacks along the *c*-axis direction, forming a three-dimensional network.

### S2. Synthesis and crystallization

The starting material 7-bromo-2-(3-fluoro­phenyl)-1-(methyl­sulfanyl)naphtho­[2,1-*b*]furan was prepared by literature method (Choi *et al.*, 1999). 3-Chloro­per­oxy­benzoic acid (77%, 224 mg, 1.0 mmol) was added in small portions to a stirred solution of the starting material (355 mg, 0.9 mmol) in di­chloro­methane (30 mL) at 273 K. After being stirred at room temperature for 4 h, the mixture was washed with saturated sodium bicarbonate solution (2 x 20 mL) and the organic layer was separated, dried over Mg_2_SO_4_, filtered and concentrated at reduced pressure. The residue was purified by column chromatography (hexane–ethyl acetate, 1:1 *v*/*v*) to afford the title compound as a colorless solid [yield 71% (258 mg); M.pt: 483–484 K; *R*_f_ = 0.48 (hexane–ethyl acetate, 1:1 *v*/*v*)]. Single crystals suitable for X-ray diffraction were prepared by slow evaporation of an acetone solution (15 mL) of the title compound (23 mg) held at room temperature.

### S3. Refinement

All H atoms were positioned geometrically and refined using a riding model with C—H = 0.95 Å for aryl and 0.98 Å for methyl H atoms, and with *U*_iso_ (H) = 1.2*U*_eq_ (C) for aryl and 1.5*U*_eq_ (C) for methyl H atoms. The positions of methyl hydrogens were optimized using the SHELXL-97 command AFIX 137 (Sheldrick, 2008). The F1 atom of the 3-fluoro­benzene ring is disordered over two positions with site occupancy factors, from refinement, of 0.851 (3) (part A) and 0.149 (3) (part B). For the proper treatment of H-atoms, carbon atoms C15 and C17 were divided with equalized coordinates and displacement parameters. The distance of equivalent C—F pairs were restrained to 1.330 (5) Å using command DFIX, and displacement ellipsoids of F1 set were restrained to be approximately spherical using the ISOR command (parameter = 0.01).

### Crystal data

**Table:**

C_19_H_12_BrFO_2_S	*F*(000) = 808
*M**_r_* = 403.26	*D*_x_ = 1.741 Mg m^−^^3^
Monoclinic, *P*2_1_/*c*	Melting point = 484 K–483 K K
Hall symbol: -P 2ybc	Mo *K*α radiation, λ = 0.71073 Å
*a* = 6.1340 (1) Å	Cell parameters from 5530 reflections
*b* = 23.0602 (5) Å	θ = 2.6–28.3°
*c* = 10.8806 (2) Å	µ = 2.83 mm^−^^1^
β = 91.166 (1)°	*T* = 173 K
*V* = 1538.76 (5) Å^3^	Block, colourless
*Z* = 4	0.74 × 0.45 × 0.38 mm

### Data collection

**Table:**

Bruker SMART APEXII CCD diffractometer	3834 independent reflections
Radiation source: rotating anode	3033 reflections with *I* > 2σ(*I*)
Graphite multilayer monochromator	*R*_int_ = 0.047
Detector resolution: 10.0 pixels mm^-1^	θ_max_ = 28.4°, θ_min_ = 1.8°
φ and ω scans	*h* = −8→8
Absorption correction: multi-scan (*SADABS*; Bruker, 2009)	*k* = −30→19
*T*_min_ = 0.229, *T*_max_ = 0.413	*l* = −14→11
14935 measured reflections	

### Refinement

**Table:**

Refinement on *F*^2^	Primary atom site location: structure-invariant direct methods
Least-squares matrix: full	Secondary atom site location: difference Fourier map
*R*[*F*^2^ > 2σ(*F*^2^)] = 0.037	Hydrogen site location: difference Fourier map
*wR*(*F*^2^) = 0.098	H-atom parameters constrained
*S* = 1.04	*w* = 1/[σ^2^(*F*_o_^2^) + (0.0539*P*)^2^ + 0.2694*P*] where *P* = (*F*_o_^2^ + 2*F*_c_^2^)/3
3834 reflections	(Δ/σ)_max_ = 0.001
228 parameters	Δρ_max_ = 0.47 e Å^−^^3^
14 restraints	Δρ_min_ = −0.91 e Å^−^^3^

### Special details

**Table:**

Experimental. ^1^H NMR (δ p.p.m., CDCl_3_, 400 Hz): 8.08-8.13 (m, 1H), 7.67-7.87 (m, 5H), 7.48-7.55 (m, 2H), 7.19-7.24 (m, 1H), 3.07 (s, 3H).
Geometry. All esds (except the esd in the dihedral angle between two l.s. planes) are estimated using the full covariance matrix. The cell esds are taken into account individually in the estimation of esds in distances, angles and torsion angles; correlations between esds in cell parameters are only used when they are defined by crystal symmetry. An approximate (isotropic) treatment of cell esds is used for estimating esds involving l.s. planes.
Refinement. Refinement of F^2^ against ALL reflections. The weighted R-factor wR and goodness of fit S are based on F^2^, conventional R-factors R are based on F, with F set to zero for negative F^2^. The threshold expression of F^2^ > 2sigma(F^2^) is used only for calculating R-factors(gt) etc. and is not relevant to the choice of reflections for refinement. R-factors based on F^2^ are statistically about twice as large as those based on F, and R- factors based on ALL data will be even larger.

### Fractional atomic coordinates and isotropic or equivalent isotropic displacement parameters (Å^2^)

**Table:**

	*x*	*y*	*z*	*U*_iso_*/*U*_eq_	Occ. (<1)
Br1	−0.54910 (4)	0.387149 (11)	−0.05723 (2)	0.03031 (11)	
S1	0.42612 (9)	0.27925 (2)	0.34599 (5)	0.01784 (14)	
O1	0.5270 (3)	0.44696 (6)	0.37648 (14)	0.0183 (3)	
O2	0.3848 (3)	0.25673 (7)	0.21917 (14)	0.0241 (4)	
C1	0.4108 (4)	0.35599 (9)	0.34160 (19)	0.0149 (4)	
C2	0.2686 (4)	0.39476 (9)	0.2724 (2)	0.0146 (5)	
C3	0.0780 (4)	0.38989 (9)	0.1956 (2)	0.0153 (5)	
C4	−0.0172 (4)	0.33718 (9)	0.1568 (2)	0.0184 (5)	
H4	0.0479	0.3016	0.1821	0.022*	
C5	−0.2012 (4)	0.33605 (10)	0.0835 (2)	0.0213 (5)	
H5	−0.2627	0.3002	0.0574	0.026*	
C6	−0.2975 (4)	0.38870 (9)	0.0474 (2)	0.0204 (5)	
C7	−0.2122 (4)	0.44064 (10)	0.0813 (2)	0.0202 (5)	
H7	−0.2804	0.4756	0.0548	0.024*	
C8	−0.0216 (4)	0.44276 (9)	0.1562 (2)	0.0173 (5)	
C9	0.0723 (4)	0.49734 (10)	0.1878 (2)	0.0208 (5)	
H9	0.0030	0.5318	0.1595	0.025*	
C10	0.2587 (4)	0.50152 (10)	0.2573 (2)	0.0206 (5)	
H10	0.3233	0.5379	0.2767	0.025*	
C11	0.3494 (4)	0.44945 (9)	0.2984 (2)	0.0169 (5)	
C12	0.5593 (4)	0.38974 (9)	0.4033 (2)	0.0166 (5)	
C13	0.7303 (4)	0.37854 (9)	0.4959 (2)	0.0170 (5)	
C14	0.9160 (4)	0.41338 (10)	0.4996 (2)	0.0187 (5)	
H14	0.9354	0.4434	0.4410	0.022*	
C15A	1.0699 (4)	0.40315 (11)	0.5903 (2)	0.0253 (6)	0.851 (3)
F1A	1.2485 (3)	0.43617 (7)	0.59705 (16)	0.0334 (5)	0.851 (3)
C15B	1.0699 (4)	0.40315 (11)	0.5903 (2)	0.0253 (6)	0.15
H15B	1.1971	0.4267	0.5927	0.030*	0.149 (3)
C16	1.0499 (4)	0.36077 (11)	0.6776 (2)	0.0278 (6)	
H16	1.1607	0.3544	0.7384	0.033*	
C17A	0.8625 (4)	0.32773 (11)	0.6738 (2)	0.0273 (6)	0.851 (3)
H17A	0.8433	0.2984	0.7340	0.033*	0.851 (3)
C17B	0.8625 (4)	0.32773 (11)	0.6738 (2)	0.0273 (6)	0.15
F1B	0.8601 (17)	0.2943 (4)	0.7701 (6)	0.034 (3)	0.149 (3)
C18	0.7026 (4)	0.33614 (10)	0.5852 (2)	0.0204 (5)	
H18	0.5741	0.3131	0.5850	0.025*	
C19	0.1839 (4)	0.26492 (10)	0.4306 (2)	0.0236 (5)	
H19A	0.0615	0.2866	0.3941	0.035*	
H19B	0.2065	0.2770	0.5163	0.035*	
H19C	0.1514	0.2233	0.4276	0.035*	

### Atomic displacement parameters (Å^2^)

**Table:**

	*U*^11^	*U*^22^	*U*^33^	*U*^12^	*U*^13^	*U*^23^
Br1	0.02564 (16)	0.03656 (18)	0.02825 (17)	−0.00225 (11)	−0.01109 (11)	0.00308 (11)
S1	0.0209 (3)	0.0138 (3)	0.0187 (3)	0.0028 (2)	−0.0025 (2)	−0.0023 (2)
O1	0.0201 (8)	0.0164 (8)	0.0184 (8)	−0.0024 (6)	−0.0026 (7)	−0.0005 (6)
O2	0.0306 (10)	0.0204 (8)	0.0213 (9)	0.0033 (7)	−0.0002 (7)	−0.0066 (7)
C1	0.0173 (11)	0.0149 (10)	0.0127 (11)	0.0003 (9)	0.0011 (8)	−0.0005 (8)
C2	0.0183 (12)	0.0142 (10)	0.0114 (11)	−0.0001 (8)	0.0016 (9)	−0.0015 (8)
C3	0.0174 (11)	0.0181 (11)	0.0105 (10)	0.0019 (8)	0.0015 (9)	−0.0012 (8)
C4	0.0219 (12)	0.0151 (11)	0.0183 (11)	−0.0002 (9)	−0.0008 (9)	0.0002 (9)
C5	0.0219 (13)	0.0218 (12)	0.0202 (12)	−0.0023 (10)	−0.0017 (9)	−0.0018 (10)
C6	0.0180 (12)	0.0287 (13)	0.0144 (11)	0.0000 (10)	−0.0023 (9)	−0.0002 (9)
C7	0.0220 (12)	0.0228 (12)	0.0156 (11)	0.0043 (10)	−0.0017 (9)	0.0031 (9)
C8	0.0211 (12)	0.0186 (11)	0.0123 (11)	0.0025 (9)	0.0003 (8)	0.0006 (9)
C9	0.0307 (14)	0.0140 (11)	0.0175 (11)	0.0038 (9)	−0.0011 (10)	0.0011 (9)
C10	0.0290 (13)	0.0138 (11)	0.0191 (12)	−0.0013 (9)	0.0025 (10)	−0.0003 (9)
C11	0.0171 (11)	0.0195 (11)	0.0141 (11)	−0.0009 (9)	0.0004 (9)	0.0009 (9)
C12	0.0189 (12)	0.0157 (11)	0.0151 (11)	0.0012 (9)	0.0025 (9)	0.0014 (8)
C13	0.0172 (12)	0.0191 (11)	0.0149 (11)	0.0017 (9)	0.0002 (9)	−0.0045 (9)
C14	0.0202 (12)	0.0184 (11)	0.0175 (12)	0.0003 (9)	0.0015 (9)	−0.0047 (9)
C15A	0.0181 (13)	0.0272 (13)	0.0304 (14)	−0.0002 (10)	−0.0025 (10)	−0.0156 (11)
F1A	0.0217 (10)	0.0360 (10)	0.0424 (11)	−0.0107 (8)	−0.0036 (8)	−0.0071 (8)
C15B	0.0181 (13)	0.0272 (13)	0.0304 (14)	−0.0002 (10)	−0.0025 (10)	−0.0156 (11)
C16	0.0300 (15)	0.0291 (14)	0.0240 (13)	0.0119 (11)	−0.0108 (11)	−0.0091 (11)
C17A	0.0320 (15)	0.0277 (13)	0.0219 (13)	0.0057 (11)	−0.0043 (11)	−0.0012 (11)
C17B	0.0320 (15)	0.0277 (13)	0.0219 (13)	0.0057 (11)	−0.0043 (11)	−0.0012 (11)
F1B	0.040 (6)	0.037 (5)	0.024 (5)	−0.005 (4)	−0.017 (4)	0.000 (4)
C18	0.0218 (13)	0.0219 (12)	0.0176 (12)	−0.0013 (10)	−0.0001 (9)	−0.0003 (9)
C19	0.0306 (14)	0.0180 (11)	0.0222 (13)	−0.0029 (10)	0.0011 (10)	0.0005 (10)

### Geometric parameters (Å, º)

**Table:**

Br1—C6	1.899 (2)	C9—C10	1.361 (3)
S1—O2	1.4912 (16)	C9—H9	0.9500
S1—C1	1.773 (2)	C10—C11	1.393 (3)
S1—C19	1.795 (2)	C10—H10	0.9500
O1—C12	1.365 (2)	C12—C13	1.463 (3)
O1—C11	1.369 (3)	C13—C18	1.391 (3)
C1—C12	1.364 (3)	C13—C14	1.393 (3)
C1—C2	1.449 (3)	C14—C15A	1.373 (3)
C2—C11	1.382 (3)	C14—H14	0.9500
C2—C3	1.428 (3)	C15A—F1A	1.335 (3)
C3—C4	1.409 (3)	C15A—C16	1.370 (4)
C3—C8	1.426 (3)	C16—C17A	1.379 (4)
C4—C5	1.369 (3)	C16—H16	0.9500
C4—H4	0.9500	C17A—C18	1.375 (3)
C5—C6	1.403 (3)	C17A—H17A	0.9500
C5—H5	0.9500	C18—H18	0.9500
C6—C7	1.355 (3)	C19—H19A	0.9800
C7—C8	1.412 (3)	C19—H19B	0.9800
C7—H7	0.9500	C19—H19C	0.9800
C8—C9	1.424 (3)		
			
O2—S1—C1	108.36 (10)	C9—C10—H10	121.9
O2—S1—C19	106.48 (11)	C11—C10—H10	121.9
C1—S1—C19	98.85 (11)	O1—C11—C2	111.47 (19)
C12—O1—C11	106.47 (17)	O1—C11—C10	122.9 (2)
C12—C1—C2	107.00 (18)	C2—C11—C10	125.6 (2)
C12—C1—S1	121.46 (17)	C1—C12—O1	110.71 (19)
C2—C1—S1	131.36 (16)	C1—C12—C13	134.5 (2)
C11—C2—C3	118.51 (19)	O1—C12—C13	114.64 (18)
C11—C2—C1	104.32 (19)	C18—C13—C14	119.8 (2)
C3—C2—C1	137.10 (19)	C18—C13—C12	120.6 (2)
C4—C3—C8	118.3 (2)	C14—C13—C12	119.5 (2)
C4—C3—C2	124.92 (19)	C15A—C14—C13	118.1 (2)
C8—C3—C2	116.73 (19)	C15A—C14—H14	120.9
C5—C4—C3	121.5 (2)	C13—C14—H14	120.9
C5—C4—H4	119.3	F1A—C15A—C16	117.0 (2)
C3—C4—H4	119.3	F1A—C15A—C14	119.6 (2)
C4—C5—C6	119.0 (2)	C16—C15A—C14	123.4 (2)
C4—C5—H5	120.5	C15A—C16—C17A	117.4 (2)
C6—C5—H5	120.5	C15A—C16—H16	121.3
C7—C6—C5	122.0 (2)	C17A—C16—H16	121.3
C7—C6—Br1	118.98 (17)	C18—C17A—C16	121.7 (2)
C5—C6—Br1	118.93 (17)	C18—C17A—H17A	119.2
C6—C7—C8	119.9 (2)	C16—C17A—H17A	119.2
C6—C7—H7	120.1	C17A—C18—C13	119.6 (2)
C8—C7—H7	120.1	C17A—C18—H18	120.2
C7—C8—C9	119.8 (2)	C13—C18—H18	120.2
C7—C8—C3	119.3 (2)	S1—C19—H19A	109.5
C9—C8—C3	121.0 (2)	S1—C19—H19B	109.5
C10—C9—C8	121.9 (2)	H19A—C19—H19B	109.5
C10—C9—H9	119.1	S1—C19—H19C	109.5
C8—C9—H9	119.1	H19A—C19—H19C	109.5
C9—C10—C11	116.3 (2)	H19B—C19—H19C	109.5
			
O2—S1—C1—C12	−136.65 (19)	C12—O1—C11—C2	−1.1 (3)
C19—S1—C1—C12	112.6 (2)	C12—O1—C11—C10	175.8 (2)
O2—S1—C1—C2	37.8 (2)	C3—C2—C11—O1	177.72 (19)
C19—S1—C1—C2	−73.0 (2)	C1—C2—C11—O1	0.1 (2)
C12—C1—C2—C11	0.9 (2)	C3—C2—C11—C10	1.0 (4)
S1—C1—C2—C11	−174.17 (18)	C1—C2—C11—C10	−176.6 (2)
C12—C1—C2—C3	−176.0 (3)	C9—C10—C11—O1	−175.0 (2)
S1—C1—C2—C3	9.0 (4)	C9—C10—C11—C2	1.4 (4)
C11—C2—C3—C4	177.0 (2)	C2—C1—C12—O1	−1.6 (3)
C1—C2—C3—C4	−6.4 (4)	S1—C1—C12—O1	174.03 (15)
C11—C2—C3—C8	−3.0 (3)	C2—C1—C12—C13	173.1 (2)
C1—C2—C3—C8	173.5 (2)	S1—C1—C12—C13	−11.2 (4)
C8—C3—C4—C5	−0.3 (3)	C11—O1—C12—C1	1.7 (3)
C2—C3—C4—C5	179.7 (2)	C11—O1—C12—C13	−174.20 (19)
C3—C4—C5—C6	−0.6 (4)	C1—C12—C13—C18	−34.8 (4)
C4—C5—C6—C7	1.0 (4)	O1—C12—C13—C18	139.7 (2)
C4—C5—C6—Br1	178.65 (18)	C1—C12—C13—C14	149.5 (3)
C5—C6—C7—C8	−0.6 (4)	O1—C12—C13—C14	−35.9 (3)
Br1—C6—C7—C8	−178.20 (17)	C18—C13—C14—C15A	2.0 (3)
C6—C7—C8—C9	177.6 (2)	C12—C13—C14—C15A	177.7 (2)
C6—C7—C8—C3	−0.3 (4)	C13—C14—C15A—F1A	−179.3 (2)
C4—C3—C8—C7	0.7 (3)	C13—C14—C15A—C16	−0.4 (4)
C2—C3—C8—C7	−179.2 (2)	F1A—C15A—C16—C17A	177.9 (2)
C4—C3—C8—C9	−177.1 (2)	C14—C15A—C16—C17A	−1.1 (4)
C2—C3—C8—C9	2.9 (3)	C15A—C16—C17A—C18	0.9 (4)
C7—C8—C9—C10	−178.4 (2)	C16—C17A—C18—C13	0.7 (4)
C3—C8—C9—C10	−0.6 (4)	C14—C13—C18—C17A	−2.2 (3)
C8—C9—C10—C11	−1.6 (3)	C12—C13—C18—C17A	−177.8 (2)

### Hydrogen-bond geometry (Å, º)

**Table:**

*D*—H···*A*	*D*—H	H···*A*	*D*···*A*	*D*—H···*A*
C18—H18···O2^i^	0.95	2.48	3.260 (3)	139
C19—H19*B*···O2^i^	0.98	2.56	3.387 (3)	142

Symmetry code: (i) *x*, −*y*+1/2, *z*+1/2.
