# Supplementary figures and images for: Crystal structure of 7-bromo-2-(3-fluoro­phen­yl)-1-(methyl­sulfin­yl)naphtho[2,1-b]furan
Source: Acta Crystallogr Sect E Struct Rep Online. 2014 Aug 13;70(Pt 9):o1001–2. doi: 10.1107/S160053681401808X (PMC4186200; doi:10.1107/S160053681401808X)

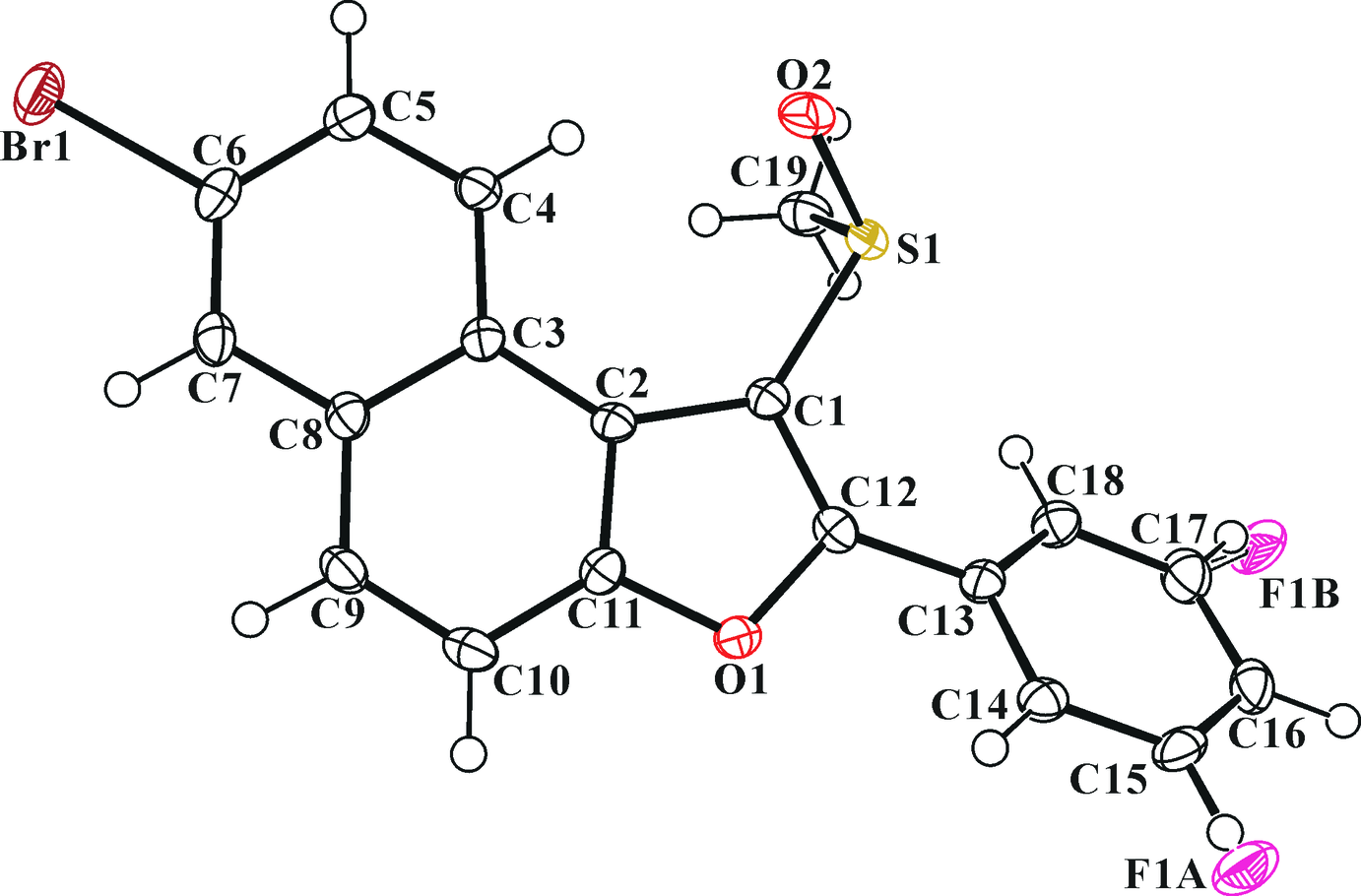

Supplement: Supplementary file 4 [file e-70-o1001-fig1.tif]

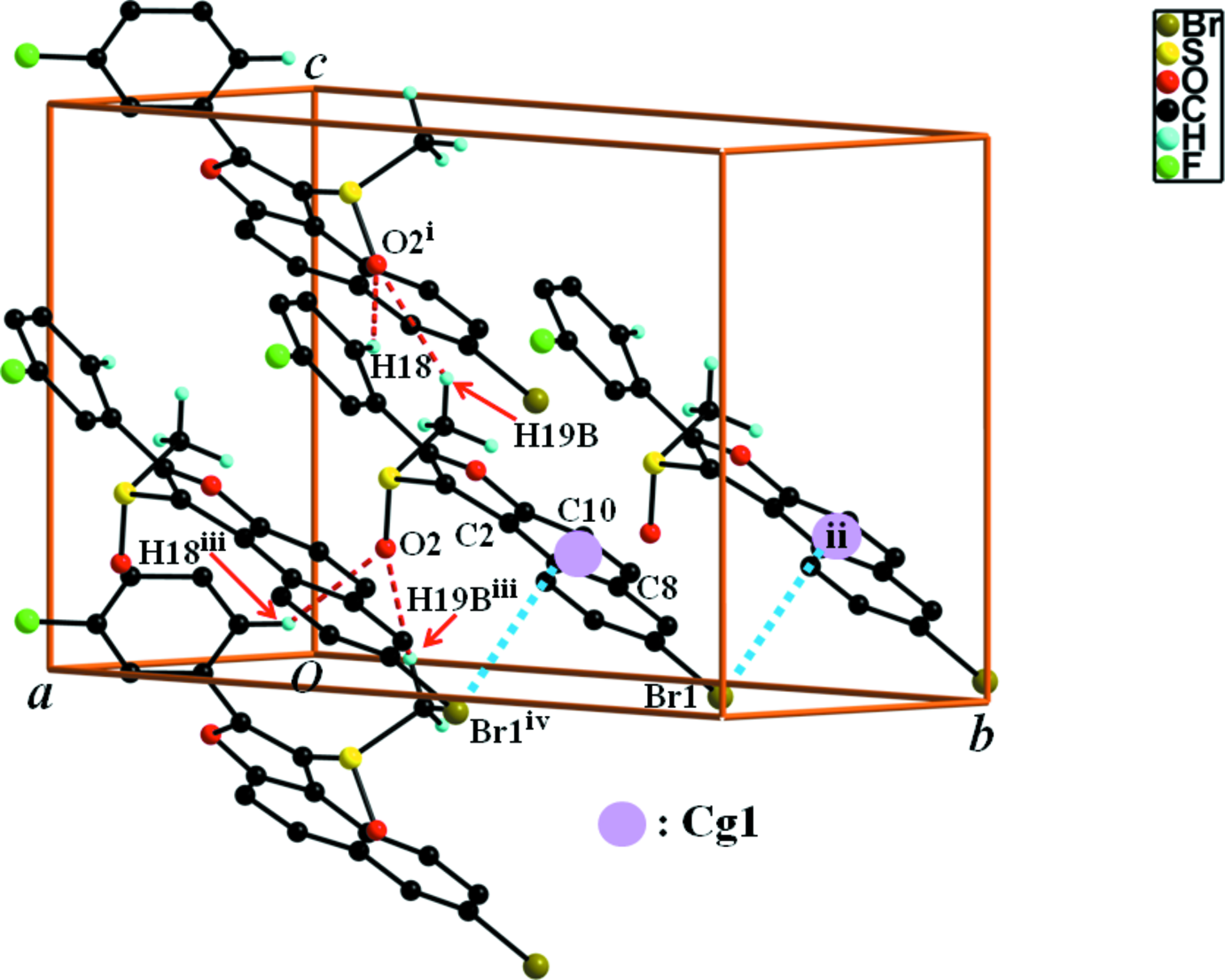

Supplement: Supplementary file 5 [file e-70-o1001-fig2.tif]
